# Electric Field-Enhanced SERS Detection Using MoS_2_-Coated Patterned Si Substrate with Micro-Pyramid Pits

**DOI:** 10.3390/nano14221852

**Published:** 2024-11-20

**Authors:** Tsung-Shine Ko, Hsiang-Yu Hsieh, Chi Lee, Szu-Hung Chen, Wei-Chun Chen, Wei-Lin Wang, Yang-Wei Lin, Sean Wu

**Affiliations:** 1 Department of Electronic Engineering, National Changhua University of Education, No. 2, Shi-Da Road, Changhua 50074, Taiwan; tsko@cc.ncue.edu.tw (T.-S.K.); victor9005@gmail.com (H.-Y.H.); chilee2001@gmail.com (C.L.); 2 Taiwan Semiconductor Research Institute, No. 26, Prosperity Road 1, Hsinchu Science Park, Hsinchu 300091, Taiwan; shchen168@narlabs.org.tw; 3 National Applied Research Laboratories, Taiwan Instrument Research Institute, 20, R&D Rd. VI, Hsinchu Science Park, Hsinchu 300092, Taiwan; weichun@narlabs.org.tw (W.-C.C.); wwl@narlabs.org.tw (W.-L.W.); 4 Department of Chemistry, National Changhua University of Education, No. 1, Jinde Road, Changhua 50074, Taiwan; linywjerry@cc.ncue.edu.tw; 5 Department of Semiconductor Engineering, Lunghwa University of Science and Technology, No. 300, Sec. 1, Wanshou Rd., Guishan District, Taoyuan City 333326, Taiwan

**Keywords:** SERS, MoS_2_, electrode, molecular aggregation

## Abstract

This study utilized semiconductor processing techniques to fabricate patterned silicon (Si) substrates with arrays of inverted pyramid-shaped micro-pits by etching. Molybdenum trioxide (MoO_3_) was then deposited on these patterned Si substrates using a thermal evaporation system, followed by two-stage sulfurization in a high-temperature furnace to grow MoS_2_ thin films consisting of only a few atomic layers. During the dropwise titration of Rhodamine 6G (R6G) solution, a longitudinal electric field was applied using a Keithley 2400 (Cleveland, OH, USA) source meter. Raman mapping revealed that under a 100 mV condition, the analyte R6G molecules were effectively confined within the pits. Due to its two-dimensional structure, MoS_2_ provides a high surface area and supports a surface-enhanced Raman scattering (SERS) charge transfer mechanism. The SERS results demonstrated that the intensity in the pits of the few-layer MoS_2_/patterned Si SERS substrate was approximately 274 times greater compared to planar Si, with a limit of detection reaching 10^−5^ M. The experimental results confirm that this method effectively resolves the issue of random distribution of analyte molecules during droplet evaporation, thereby enhancing detection sensitivity and stability.

## 1. Introduction

Surface-enhanced Raman spectroscopy (SERS) has recently emerged as a highly popular detection technology [1,2,3]. The intensity of its signal depends on the surface morphology of the substrate as well as the shape and size of the materials used [4,5,6]. Due to its non-destructive nature and high sensitivity, SERS has found widespread applications in biological detection and chemical analysis, making it a subject of intense research [7,8]. The enhancement mechanisms of SERS can be broadly categorized into electromagnetic and chemical enhancement mechanisms [9,10]. Recently, two-dimensional (2D) materials that leverage chemical enhancement mechanisms have also played a significant role in Raman spectroscopy. These materials further enhance the SERS signals from substrates, and as they transition from bulk materials to a few layers or even monolayers, their bandgap changes from an indirect to a direct gap [11,12,13,14], thereby increasing the number of electronic transition pathways and the probability of electron transitions. Research conducted by Xiuju Song et al. demonstrated that 2D materials such as NbS_2_, MoS_2_, and graphene perform well as substrates for fast and reliable SERS detection [15]. Ge Song et al. employed WO_3_ as a material for SERS enhancement, where the ultra-thin WO_3_ not only alters the surface morphology of the substrate but also offers more pathways for electron migration [16]. Combining these advantages, we previously achieved a 75-fold enhancement in the SERS signal by covering silicon nanocolumn substrates with MoS_2_ thin films [17].

One major challenge in applying current SERS technology is the inability to effectively aggregate target molecules at specific regions of the substrate. As a result, analyte molecules are distributed randomly over the detection surface, making it difficult to control signal uniformity while increasing the time required to identify target molecules, thus reducing the overall detection efficiency [18]. While traditional SERS substrates utilizing noble metals, such as gold and silver nanostructures, are highly effective in generating hot spots for signal enhancement, they come with several disadvantages. In addition to the cost and the risk of carbonization of analytes, another significant issue is the possibility of overheating at hot spots. This overheating can lead to thermal degradation or destruction of the analyte molecules, compromising the accuracy and reliability of the SERS analysis. Similarly, 2D materials that utilize charge transfer chemical mechanisms also exhibit this issue. Therefore, effectively controlling molecular distribution and aggregation remains a critical challenge in SERS technology. Research by H. Lüth et al. demonstrated that a capacitor structure formed by an electrolyte–insulator–semiconductor system exhibits high sensitivity and stability in molecular detection. They used a porous silicon substrate as a base layer, covered it with electrodes, and deposited a Si_3_N_4_ film on top, allowing penicillinase to be physically adsorbed onto the capacitor, which showed promising detection performance [19]. Silicon, as a well-established semiconductor material, can be processed using lithography to generate various surface structures, making it highly suitable as a SERS substrate for biomedical detection [20].

To address the problem of uneven analyte distribution, this study employed semiconductor processing techniques to fabricate patterned silicon substrates with inverted pyramid-shaped micron-scale pore arrays, followed by the deposition of a few atomic layers of MoS_2_. MoS_2_, with its high surface area due to its 2D structure and its ability to enhance SERS signals through a charge transfer mechanism, holds significant potential as an enhancing material. During the experiments, we applied a longitudinal electric field to effectively direct Rhodamine 6G (R6G) molecules into the pits, thereby solving the issue of random distribution of molecules during droplet evaporation. SERS analysis showed that the signal intensity within the pits of the substrate was approximately 274 times higher that of the planar silicon substrate, with a limit of detection (LOD) of 10^−5^ M. The LOD of 10^−5^ M achieved by our MoS_2_/patterned Si SERS substrate is considered satisfactory, particularly in the context of chemical enhancement mechanisms using 2D materials, for which it is generally more challenging to achieve high sensitivities compared to conventional SERS substrates based on precious metals like Au and Ag. These findings confirm that the proposed method significantly enhances the sensitivity and stability of the SERS substrate, providing an effective strategy to address the challenges of random molecular distribution and to improve detection efficiency.

## 2. Materials and Methods

**Figure 1:**
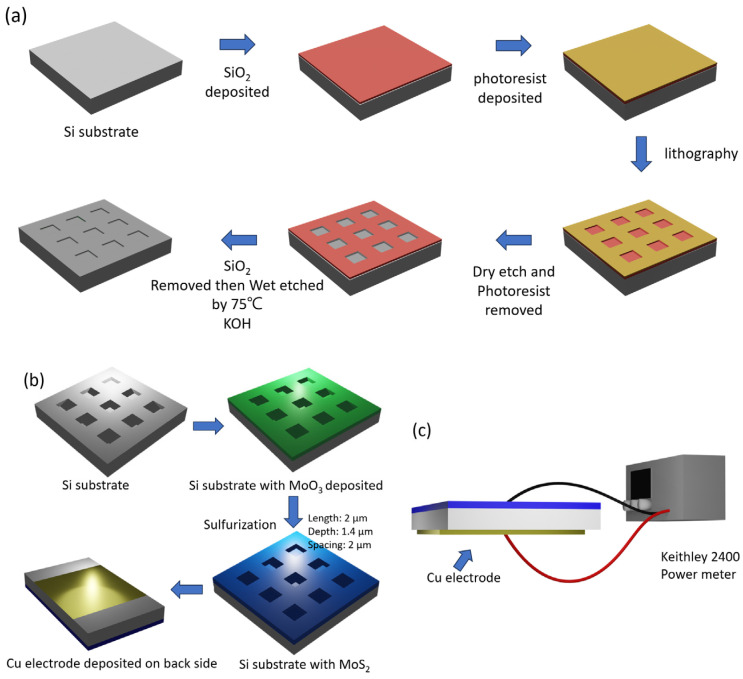
(**a**) Lithography process flowchart. (**b**) Fabrication of the SERS substrate: Si substrate patterned with inverted pyramid pits, MoO_3_ deposition, sulfurization to MoS_2_, and Cu electrode deposition. (**c**) SERS measurement setup: Cu electrodes are connected to a Keithley 2400 source meter to apply a longitudinal electric field for R6G localization.

We initially deposited a layer of silicon dioxide (SiO_2_) onto a silicon substrate as a hard mask. Photoresist was then spin-coated onto the substrate, followed by an exposure process. Subsequently, plasma etching was used to remove the SiO_2_ layer, and the photoresist was stripped away. The sample was then immersed in a 47% KOH solution at 75 °C for etching to form inverted pyramid-shaped micron-sized pores. The full lithography process can be seen in Figure 1a. Following this, the sample was immersed in buffered oxide etch at room temperature to remove the remaining SiO_2_ layer. According to our design, the spacing between the pits was set at about 2 µm. Figure 1b shows the fabrication flow of our substrate.

After the substrate preparation, the sample was placed in a thermal evaporation system to deposit a thin layer of MoO_3_, approximately 2 nm in thickness. It was then subjected to a high-temperature furnace treatment. The chamber pressure was maintained at approximately 10^−3^ torr, while the temperature was gradually increased from 50 °C to 700 °C at a rate of 10 °C per minute. At 400 °C, the flowmeter and external chamber valves were opened to introduce a carrier gas (argon) at a flow rate of 200 sccm, allowing the reaction gases to flow across the substrate. The temperature was held at 700 °C for five minutes before being lowered at a rate of 5 °C per minute. We used scanning electron microscopy (SEM) (ULTRA plus FESEM, Oberkochen, Germany) to observe the surface morphology and cross-sectional area of the samples and Raman spectroscopy to determine the atomic layer thickness of the MoS_2_. In the SEM measurements, the sample was secured on the sample holder using conductive copper tape. After placing the sample into the SEM chamber, we captured SEM images of both the surface and side of the sample by rotating the sample stage. Due to its two-dimensional structure and large surface area, the MoS_2_ thin films are suitable as SERS substrates, enhancing surface-enhanced Raman scattering signals.

For the electrodes, copper (Cu) was selected as the material. Following the completion of the earlier steps, a 35 nm thick copper electrode was deposited with the help of a mask. Before conducting SERS measurements, different concentrations of R6G solution were drop-cast onto the patterned surface for preparation. Figure 1c illustrates the schematic for the SERS measurements. For the SERS measurements, the substrate was connected to a Keithley 2400 current-voltage source, which precisely controlled the electric field applied to the substrate. The copper electrodes on the substrate served to apply a longitudinal electric field, effectively confining the R6G molecules to the interior of the inverted pyramid-shaped pores, thereby increasing Raman signal intensity and measurement stability.

The Raman spectral measurements were performed using the Nanofinder 30 system from Tokyo Instruments (Tokyo, Japan). For the characterization of MoS_2_, a 488 nm laser was employed due to its higher photon energy, which facilitates clearer detection of atomic vibrational modes. Conversely, for the analysis of R6G, a 633 nm laser was used to minimize fluorescence interference, which is often induced at shorter wavelengths, thereby ensuring more accurate Raman spectra. Specifically, a 488 nm laser with a power output of 1 mW and a grating resolution of 1800 lines/mm was utilized for the MoS_2_ thin film analysis, whereas a 633 nm laser with a power of 0.3 mW was used for the R6G molecular detection. The laser spot size was adjusted to focus to 3 μm during the Raman spectral measurements, whereas for the Raman mapping, it was focused to approximately 1 μm. This adjustment was achieved by optimizing the position of the 100× microscope objective, which had a numerical aperture of 0.9. The laser acquisition time was set to 20 s per measurement. These conditions allowed us to accurately capture and compare the impact of the electric field on molecular aggregation.

## 3. Results and Discussion

**Figure 2:**
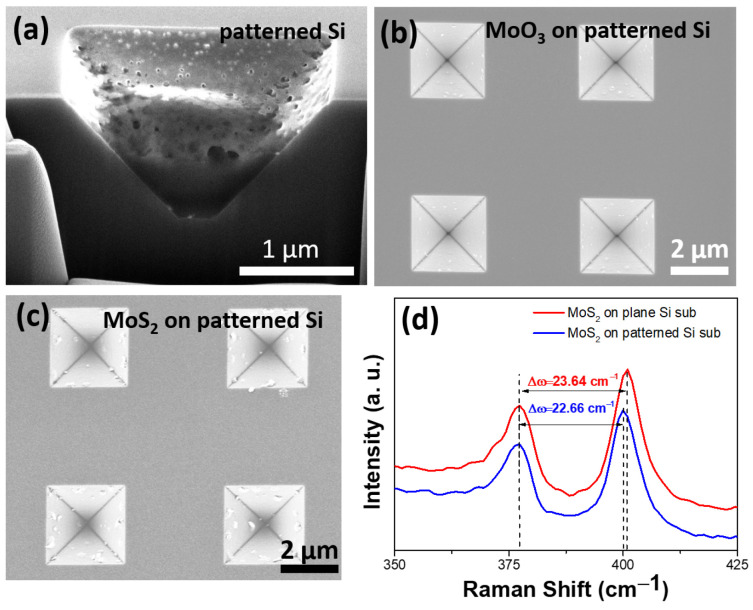
SEM images of (**a**) patterned Si, (**b**) MoO_3_ deposited on patterned Si, and (**c**) MoS_2_ formed on patterned Si. (**d**) Raman spectra of MoS_2_ on patterned Si (blue) and planar Si (red), showing characteristic MoS_2_ peaks.

Figure 2a displays a cross-sectional scanning electron microscope (SEM) image of the patterned Si substrate after KOH etching. The surface of the silicon substrate clearly shows inverted pyramid-shaped micron-sized pits composed of four (111) planes. This structure was created using a wet etching process that exploits the crystallographic anisotropy of Si, resulting in structures with a high aspect ratio [21]. Such an inverted pyramid structure provides an excellent platform for subsequent SERS applications, as these microstructures can effectively confine molecules, thereby enhancing Raman scattering signals. Figure 2b shows an SEM image of the patterned Si substrate after the deposition of MoO_3_. It is evident that MoO_3_ uniformly covers the surface of the inverted pyramid-shaped substrate. Figure 2c illustrates the surface after sulfurization in a high-temperature furnace, converting MoO_3_ to molybdenum disulfide (MoS_2_). The resulting surface appears similar to our previous study involving the growth of core-shell nanorods, with no substantial difference between the MoO_3_ and MoS_2_-coated surfaces [17,22]. Controlling the film thickness during this process is crucial for achieving optimal SERS performance, as the number of MoS_2_ layers directly influences the surface-enhanced Raman scattering effect. Therefore, we used Raman measurements to analyze the thickness of the samples and compared the growth under identical conditions on both patterned and planar Si substrates.

As shown in Figure 2d, the Raman spectrum indicates successful growth of MoS_2_ on the Si substrate using the described method. The characteristic MoS_2_ peaks at 384 cm^−1^ (E^1^_2g_ mode) and 408 cm^−1^ (A^1^_g_ mode) exhibit a Δω value of 23.64 and 22.66 cm⁻^1^ for the planar and pit regions, respectively, indicating 2–3 layers of MoS_2_ [23,24]. This result suggests that the shadowing effect created by the patterned substrate leads to fewer MoS_2_ layers in the pits, thereby affecting the crystal structure and vibrational modes of MoS_2_. As the energy band structure of MoS_2_ tends to favor a direct band gap at fewer atomic layers [11], this property enhances the charge transfer mechanism in SERS, further optimizing the intensity and sensitivity of the Raman scattering signal.

**Figure 3:**
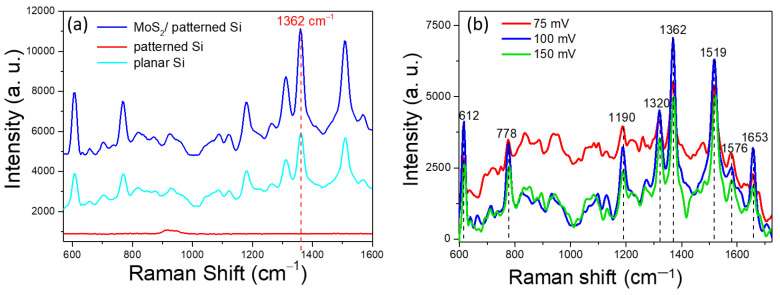
(**a**) SERS spectra for MoS_2_/patterned Si, patterned Si, and planar Si substrates with 10^−3^ M R6G, showing enhanced signal for MoS_2_/patterned Si at 1362 cm^−1^. (**b**) SERS spectra of MoS_2_/patterned Si under different voltages, with optimal enhancement at 100 mV.

After confirming the structure of the few-layer MoS_2_/patterned Si SERS substrate, we conducted a comparative SERS analysis on different substrates, including MoS_2_/patterned Si, patterned Si, and planar Si substrates. To determine the optimal voltage, we applied 10^−3^ M concentrations of R6G to each of these samples and compared the effects of different applied voltages (75, 100, and 150 mV). Figure 3a shows that the MoS_2_/patterned Si substrate exhibits significantly higher Raman signal intensity compared to the other substrates. In particular, the Raman peak at 1362 cm⁻^1^, which corresponds to the C–C stretching vibration mode of the R6G molecule, is one of its characteristic peaks [25]. By comparison, the SERS signals of the patterned Si substrate (without MoS_2_) and the planar Si substrate under the same conditions are notably weaker. This indicates that the patterned structure of the substrate enhances the confinement of R6G molecules, thereby increasing the interaction between the analyte and the substrate. The presence of MoS_2_ on the patterned substrate further enhances the Raman signal intensity, primarily due to the unique characteristics of the MoS_2_ 2D structure and its role in the charge transfer effect within the chemical enhancement mechanism. Specifically, MoS_2_ acts as an efficient charge mediator, facilitating electron transfer between the analyte molecule, R6G, and the substrate. Upon laser excitation, R6G molecules can undergo photo-induced electron transfer to MoS_2_, which, due to its direct band gap and favorable electronic structure, effectively stabilizes these electrons before transferring them back. This cyclic CT process significantly enhances the Raman signal of the adsorbed molecules. The high surface area of the MoS_2_ also provides abundant active sites for the adsorption of R6G, ensuring a more efficient coupling between the molecular electronic states and the 2D material. The presence of MoS_2_ on the patterned substrate further enhances the signal intensity, likely due to the high surface area of the MoS_2_ 2D structure and the charge transfer effect in the chemical enhancement mechanism. These structural advantages contribute to the favorable SERS performance of the MoS_2_/patterned Si substrate compared to the other two types of substrates, resulting in enhanced Raman signal intensity and sensitivity, even if it may not match the highest levels reported for precious metal-based substrates.

Figure 3b shows the SERS results of the MoS_2_/patterned Si substrate under different applied voltages ranging from 75 mV to 150 mV. At 75 mV, the Raman signal is relatively weak, indicating that low voltage might not significantly influence the localization of analyte molecules or the interaction between the analyte and the substrate. At 100 mV, the Raman signals, particularly at 1362 cm^−1^ and 1519 cm^−1^, reach their highest intensities, suggesting that this voltage condition significantly enhances molecular localization within the substrate and effectively improves the SERS effect. Conversely, at 150 mV, the Raman signal decreases, suggesting that excessive voltage could negatively impact molecular localization, possibly pushing analyte molecules out of the substrate’s pits or leading to over-concentration in specific regions, thereby reducing the SERS signal intensity. Based on the analysis above, an applied voltage of 100 mV is found to be the optimal condition for enhancing the SERS signal of R6G molecules while maintaining stability. To further understand the distribution of R6G molecules under different voltage conditions, a Raman mapping analysis was conducted on this series of samples.

**Figure 4:**
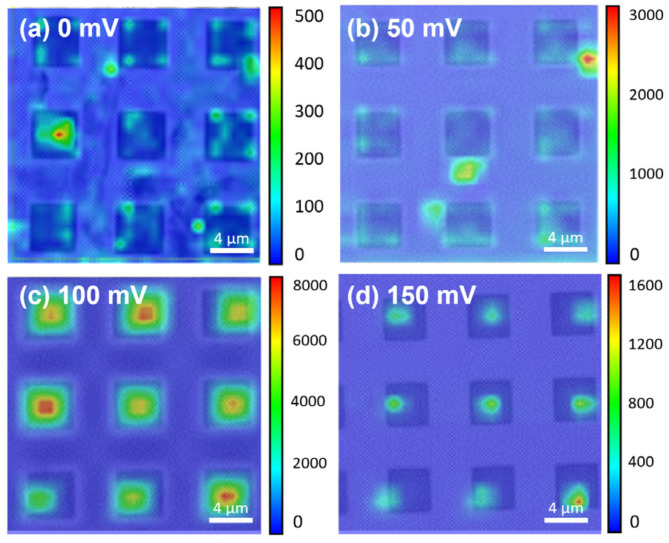
Raman mapping images of R6G molecules on MoS_2_/patterned Si SERS substrate under applied voltages: (**a**) 0 mV, (**b**) 50 mV, (**c**) 100 mV, and (**d**) 150 mV, overlaid with corresponding OM images. The color scale represents signal intensity, ranging from blue (low) to red (high). Optimal SERS enhancement and uniform localization of R6G molecules within the pits are observed at 100 mV, whereas lower or higher voltages result in diminished signal intensity and less effective molecule aggregation.

Figure 4a–d present the Raman mapping results of R6G molecules on the MoS_2_/patterned Si SERS substrate under four different applied voltages: (a) 0 mV, (b) 50 mV, (c) 100 mV, and (d) 150 mV. To provide a more comprehensive understanding of the R6G distribution, we have overlaid the optical microscopy (OM) images with the corresponding Raman mapping results. This approach offers a visual reference for both the physical structure of the pits and the intensity distribution of R6G within them, allowing readers to clearly observe where R6G molecules have aggregated. By combining these two sets of information into a single image, we provide a more efficient representation of the data, thereby enhancing the clarity of molecular localization while minimizing redundancy. These Raman mapping images are overlaid with the corresponding OM images to facilitate the identification of the R6G distribution. The color bars represent the intensity of the Raman signals, ranging from blue (low) to red (high). Analyzing these mapping images, it is evident that the applied voltage significantly influences the localization of R6G molecules and the enhancement of the SERS signal. In Figure 4a, without any applied voltage, the Raman signal distribution appears random with overall low intensity, suggesting uneven distribution of R6G molecules on the substrate, which makes effective confinement within the pit structures difficult. This random distribution further leads to reduced SERS signal intensity. In Figure 4b, applying 50 mV results in the appearance of higher signals in some pits, indicating that the electric field begins to assist in R6G accumulation, but the overall effect is still limited, and the intensity and concentration are not significantly improved. Consequently, the R6G molecules were not fully directed into the pyramid pits, leading to some molecules being distributed outside the pit structures. Figure 4c reveals a notable improvement under 100 mV; most of the pits show high-intensity regions represented by red colors, indicating that the applied voltage effectively attracts R6G molecules to the interior of the pits, forming stable local aggregation and significantly enhancing the SERS signal. This result suggests that the applied voltage of 100 mV is sufficient to create a field that facilitates the effective and stable localization of target molecules. The mapping at this voltage shows the highest uniformity and intensity among all tested conditions. However, as shown in Figure 4d, when the voltage is increased to 150 mV, the signal intensity and concentration decrease. In some pits, the signal is visibly reduced, possibly because the higher electric field expels R6G molecules from the pit or induces redistribution, leading to less stable signals. Thus, applying excessive voltage impairs the effective confinement and localization of R6G molecules, adversely affecting the uniformity and intensity of the SERS signal. These mapping results indicate that applying moderate voltage (e.g., 100 mV) significantly enhances the localization of R6G molecules, especially within the pits, thereby improving SERS intensity and uniformity. In contrast, without applied voltage or with excessive voltage, the molecules tend to distribute randomly or become redistributed, leading to diminished SERS performance. The findings confirm the importance of the electric field in controlling molecular distribution and highlight the critical role of choosing an appropriate voltage for optimizing SERS sensitivity and stability.

**Figure 5:**
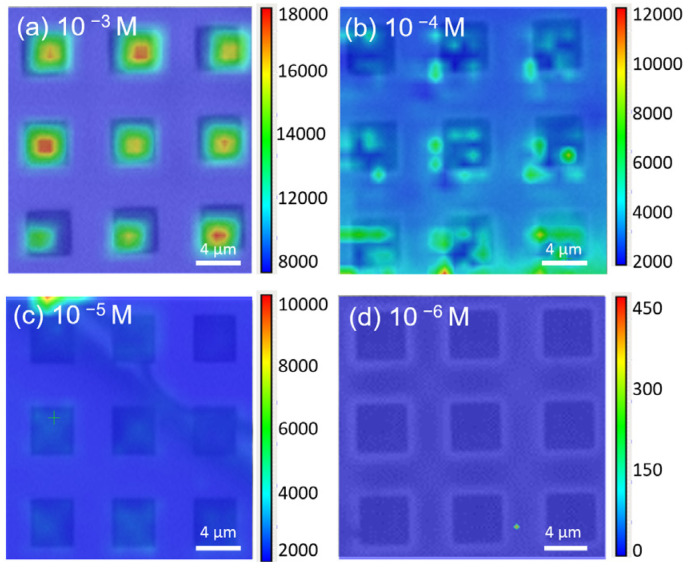
Raman mapping images of R6G on MoS_2_/patterned Si substrate at different concentrations: (**a**) 10^−3^ M, (**b**) 10^−4^ M, (**c**) 10^−5^ M, and (**d**) 10^−6^ M, overlaid with corresponding OM images. The signal intensity decreases as the concentration reduces, with strong and uniform signals observed at higher concentrations (10^−3^ M), while the signals are significantly weaker at lower concentrations, indicating the detection limit around 10^−5^ M.

Figure 5 shows the Raman mapping results of R6G solutions at different concentrations on the MoS_2_/patterned Si SERS substrate, overlaid with the optical microscopy (OM) images for comparison. The concentrations include (a) 10^−3^ M, (b) 10⁻^4^ M, (c) 10^−5^ M, and (d) 10^−6^ M. From Figure 5a–c, it can be observed that as the R6G concentration decreases from 10^−3^ M to 10^−5^ M, the Raman signal intensity gradually weakens. The applied electric field effectively induces molecular aggregation in the pits for all the tested R6G concentrations. The differences in signal intensity are primarily due to the chosen scale bar limits in the Raman mapping, standardized to enhance clarity. At 10^−3^ M, the SERS signal is strongest and uniformly distributed within the patterned Si pits, indicating that at this concentration, there are sufficient R6G molecules to be effectively captured within the substrate pits and interact with MoS_2_, thereby producing a significant enhancement effect. As the concentration decreases to 10⁻^4^ M and 10^−5^ M, the Raman signal weakens, especially at 10^−5^ M, where signals are still present but notably less intense compared to higher concentrations, although some enhancement remains. When the R6G concentration further drops to 10^−6^ M, as shown in Figure 5d, the Raman signal nearly vanishes, with only a few weak signals present, mostly at the background noise level. This suggests that at such a low concentration, the MoS_2_/patterned Si SERS substrate can no longer effectively aggregate and enhance the Raman signal from R6G molecules, indicating that the detection sensitivity reaches its limit at around 10^−5^ M.

These Raman mapping results represent a large dataset that captures the signal intensity distribution across the entire substrate region, providing a more comprehensive and representative analysis compared to previous single-point SERS measurements we conducted [26]. In past SERS studies, the calculation of the enhancement factor typically relied on signals from a single specific hotspot, making the EF results overly sensitive to certain local characteristics and lacking generalizability. In contrast, with Raman mapping, we can observe the signal distribution at multiple positions across the entire substrate, offering a better reflection of the substrate’s overall SERS performance. This approach is particularly important for substrates with non-uniform structures, like the patterned Si substrates in this study. Given the extensive data in the Raman mapping, we selected relatively strong signal points within the pits and used multi-point averaging to represent the enhancement effect in the pits. This averaging process not only eliminates extreme values caused by occasional hotspots but also reflects the overall SERS enhancement effect within the pits. By comparing the averaged Raman signal intensity of R6G within the pits to that measured on the planar Si substrate, we observed an approximately 274-fold increase in signal. These results indicate that the inverted pyramid pits on the patterned Si substrate effectively capture and concentrate the target molecules, significantly enhancing the SERS signal intensity. The importance of these findings lies in overcoming the bias introduced by previous single-point measurements, demonstrating the substrate’s comprehensive ability for molecule capture and signal enhancement across the entire SERS surface. This mapping-based analytical approach is more reliable and convincing, especially for evaluating the uniformity and stability of SERS substrates, offering a more holistic assessment framework. Thus, the enhanced signal results based on this mapping technique not only reflect the superior performance of the substrate but also provide valuable reference data for the design and application of future SERS substrates. Research into the use of external electric fields for molecular aggregation is still relatively limited, but our previous important findings can be drawn from recent studies involving different GaN planes. In particular, a-plane and c-plane GaN have been employed to investigate the role of built-in electric fields in facilitating polar molecule aggregation. The a-plane GaN substrates exhibit intrinsic polarization fields that effectively promote the aggregation of polar molecules or analytes within specific regions, which greatly enhances the SERS signal. This effect has been observed prominently with a-plane GaN due to the direction of the internal electric fields, which align favorably for aggregating molecules within its pitted structures [27]. In contrast, c-plane GaN lacks a significant polarization effect along its primary plane, resulting in a more uniform molecular distribution with less pronounced aggregation within the pits. This difference between the two substrates underscores the importance of crystal orientation in creating effective SERS hotspots. Therefore, the application of a-plane GaN, with its enhanced ability to attract and localize analytes, suggests a promising approach for improving the sensitivity and efficiency of SERS-based molecular detection systems [28].

## 4. Conclusions

This study successfully developed a high-performance SERS substrate based on a patterned Si substrate combined with MoS_2_ material. By etching a patterned Si substrate to create an array of inverted pyramid-shaped micro-scale pits and depositing few-layer MoS_2_ films, followed by applying an electric field using interdigitated copper electrodes, we performed SERS analysis. The experimental results show that, under a 100 mV applied field, R6G molecules effectively accumulated within the pits, significantly enhancing the SERS signal. The intensity in the pits was approximately 274 times higher compared to the flat Si substrate. Additionally, Raman mapping demonstrated that, even as the R6G concentration decreased from 10^−3^ M to 10^−5^ M, the substrate maintained good enhancement and uniformity of the signals. The LOD of the SERS substrate for R6G molecules was found to be 10^−5^ M, showcasing excellent sensitivity and stability, effectively addressing the issue of random molecule distribution after droplet evaporation, which otherwise causes inconsistent signals. The developed SERS substrate is advantageous due to its simple fabrication process, low cost, high sensitivity, and reproducibility, making it suitable for applications such as biomolecule detection.

## Data Availability

The data are contained within the article.
